# Endomucin inhibits VEGF-induced endothelial cell migration, growth, and morphogenesis by modulating VEGFR2 signaling

**DOI:** 10.1038/s41598-017-16852-x

**Published:** 2017-12-07

**Authors:** Cindy Park-Windhol, Yin Shan Ng, Jinling Yang, Vincent Primo, Magali Saint-Geniez, Patricia A. D’Amore

**Affiliations:** 1 Schepens Eye Research Institute/Massachusetts Eye and Ear, Boston, MA USA; 2 Department of Ophthalmology, Harvard Medical School, Boston, MA USA; 3 Department of Pathology, Harvard Medical School, Boston, MA USA

**Keywords:** Glycobiology, RNAi

## Abstract

Angiogenesis is central to both normal and pathologic processes. Endothelial cells (ECs) express O-glycoproteins that are believed to play important roles in vascular development and stability. Endomucin-1 (EMCN) is a type I O-glycosylated, sialic-rich glycoprotein, specifically expressed by venous and capillary endothelium. Evidence has pointed to a potential role for EMCN in angiogenesis but it had not been directly investigated. In this study, we examined the role of EMCN in angiogenesis by modulating EMCN levels both *in vivo* and *in vitro*. Reduction of EMCN *in vivo* led to the impairment of angiogenesis during normal retinal development *in vivo*. To determine the cellular basis of this inhibition, gain- and loss-of-function studies were performed in human retinal EC (HREC) *in vitro* by EMCN over-expression using adenovirus or EMCN gene knockdown by siRNA. We show that EMCN knockdown reduced migration, inhibited cell growth without compromising cell survival, and suppressed tube morphogenesis of ECs, whereas over-expression of EMCN led to increased migration, proliferation and tube formation. Furthermore, knockdown of EMCN suppressed VEGF-induced signaling as measured by decreased phospho-VEGFR2, phospho-ERK1/2 and phospho-p38-MAPK levels. These results suggest a novel role for EMCN as a potent regulator of angiogenesis and point to its potential as a new therapeutic target for angiogenesis-related diseases.

## Introduction

Angiogenesis, the process through which new vessels grow from existing vessels via branching morphogenesis^1^, is central to many physiological and pathological processes such as embryonic development, wound healing, tumor growth and metastasis, as well as several ocular diseases^2,3^. During angiogenesis, small blood vessels form by budding and sprouting from larger vessels, generally venules. Capillary formation involves a number of highly orchestrated steps including degradation of extracellular matrix by endothelial cells (ECs), endothelial migration into the surrounding tissue, proliferation, alignment, lumen formation, and finally anastomosis of the nascent vessel with adjacent sprouts^4–6^. These steps are regulated by an array of soluble growth factors as well as by homotypic and heterotypic cell-cell interactions^7^.

One of the key regulators of the angiogenic responses in ECs is vascular endothelial growth factor-A (VEGF). VEGF, a prototypic angiogenic factor^8,9^, has been shown to play a central role in regulation of vascular development, developmental and pathologic angiogenesis, vascular permeability, and cell survival pathways^10^. Acting primarily via VEGF receptor 2 (VEGFR2), VEGF activates the EC through signaling cascades that enable selection of a tip cell and subsequent vessel branching. VEGF-binding to VEGFR2 induces receptor dimerization and autophosphorylation, resulting in increased VEGFR2 tyrosine kinase activity and phosphorylation of additional tyrosine residues. These trigger downstream signaling cascades including p42/44 ERK1/2^11^ and PI3k/Akt^10^ promoting EC proliferation, migration, and survival.

Endomucin-1 (EMCN) is an 80–120 kDa transmembrane sialomucin that is endothelial-specific, and expressed solely on the surface of the capillary and venous, but not arterial, endothelium^12–15^. Accordingly, EMCN is robustly expressed in highly vascularized tissues such as the heart, kidney, and lung^16^. Importantly, we have identified EMCN expression to be polarized to the apical surface of the vascular endothelium, where it functions as an anti-adhesive molecule, preventing interactions between neutrophils and ECs^17^. It is a type I O-glycosylated sialic-rich glycoprotein that is rich in serine and threonine residues^12,16,18^. As a result of the O-linked glycans on the protein backbone, the molecule adopts a rigid and extended rod-like structure, contributing to its role in regulating cell-cell and cell-matrix interactions.

Several lines of evidence suggest that EMCN may play a role in angiogenesis. Expression of EMCN is increased during EC proliferation or following stimulation with tumor-conditioned media^16^ and GATA2-regulated EMCN gene expression has been suggested to be involved in vessel formation^19^. Moreover, we have previously reported that cystic embryoid bodies formed from VEGF-null murine embryonic stem cells contain ECs that lack EMCN expression and fail to organize into vessel-like structures^20^. This same study demonstrated that EMCN expression by EC was downstream of VEGF. Taken together, these observations point to a role for EMCN in vascular development.

In the present report, we show that EMCN knockdown significantly inhibited angiogenesis in a murine model of retinal vascular development. Cell-based experiments demonstrated that EMCN is involved in modulating VEGF-induced EC migration, growth, and tube morphogenesis by the modulation of VEGFR2 activation.

## Results

### EMCN is expressed by ECs in the developing mouse retinal vasculature

**Figure 1:**
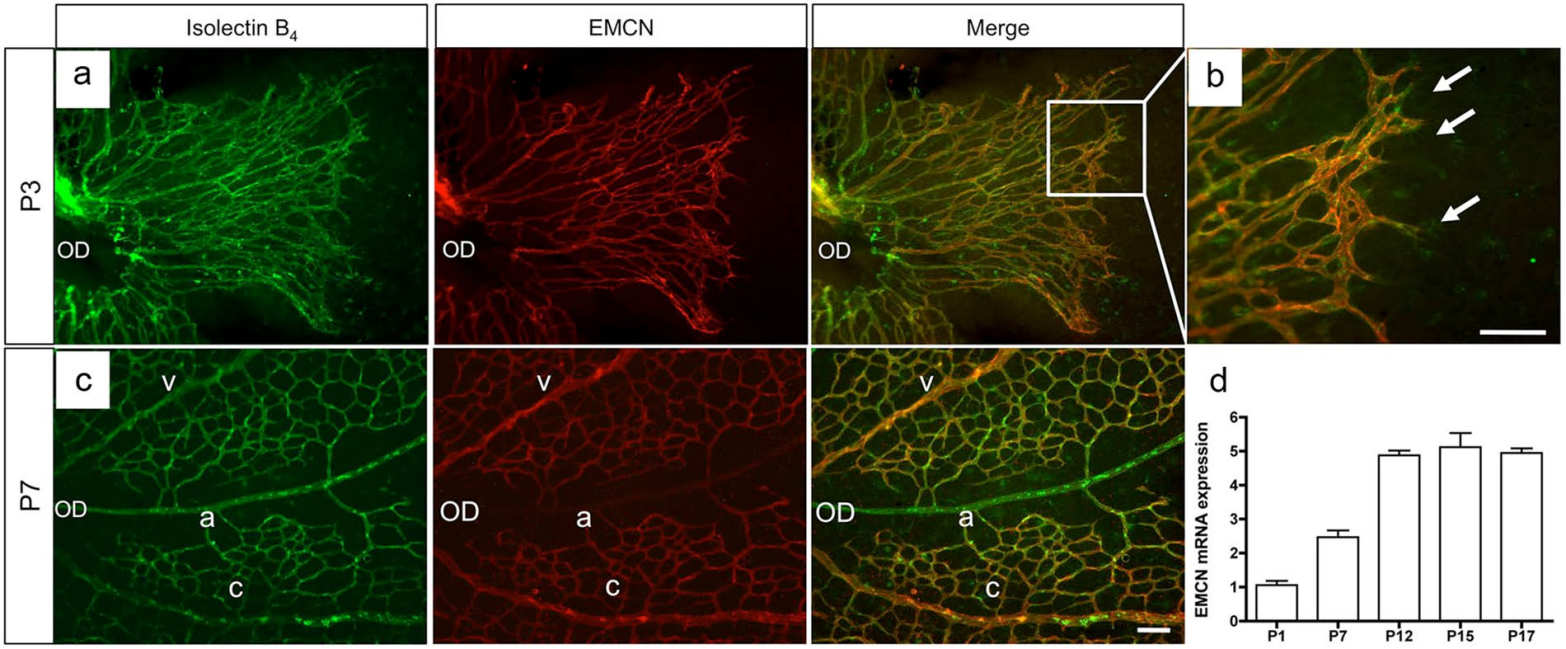
EMCN is expressed in endothelial cells in the developing mouse retinal vasculature. (**a**–**c**) Following enucleation, retinas from wild-type mice were fixed in 4% paraformaldehyde. Vessels were stained with isolectin B_4_ (green) and endomucin (EMCN; red) at (**a**,**b**) postnatal day 3 (P3) and (**c**) 7 (P7). Positive-EMCN staining localizes in veins (v) and capillaries (**c**) but not arteries (**a**). (**d**) qRT-PCR analysis of EMCN mRNA levels at different postnatal (P) ages. Arrow indicates filopodia. OD: optic disc. Scale bar, 200 μm.

To begin to investigate the role of EMCN in the development of the retinal vasculature, we characterized the expression pattern of EMCN during the postnatal vascular development of the mouse retina. The forming retinal vasculature of postnatal day (P) 3 wild-type pups was strongly labeled by staining for EMCN (Fig. 1a), which became restricted to veins and capillaries by P7 (Fig. 1c). EMCN was detected in both sprouting ECs (particularly in the filopodia) as well as in patent new vessels (Fig. 1b). Consistent with the immunohistological data, EMCN mRNA was strongly expressed starting at P1, peaked at P12, and plateaued at P17 (Fig. 1d).

### Loss of EMCN results in defective retinal vascular development

**Figure 2:**
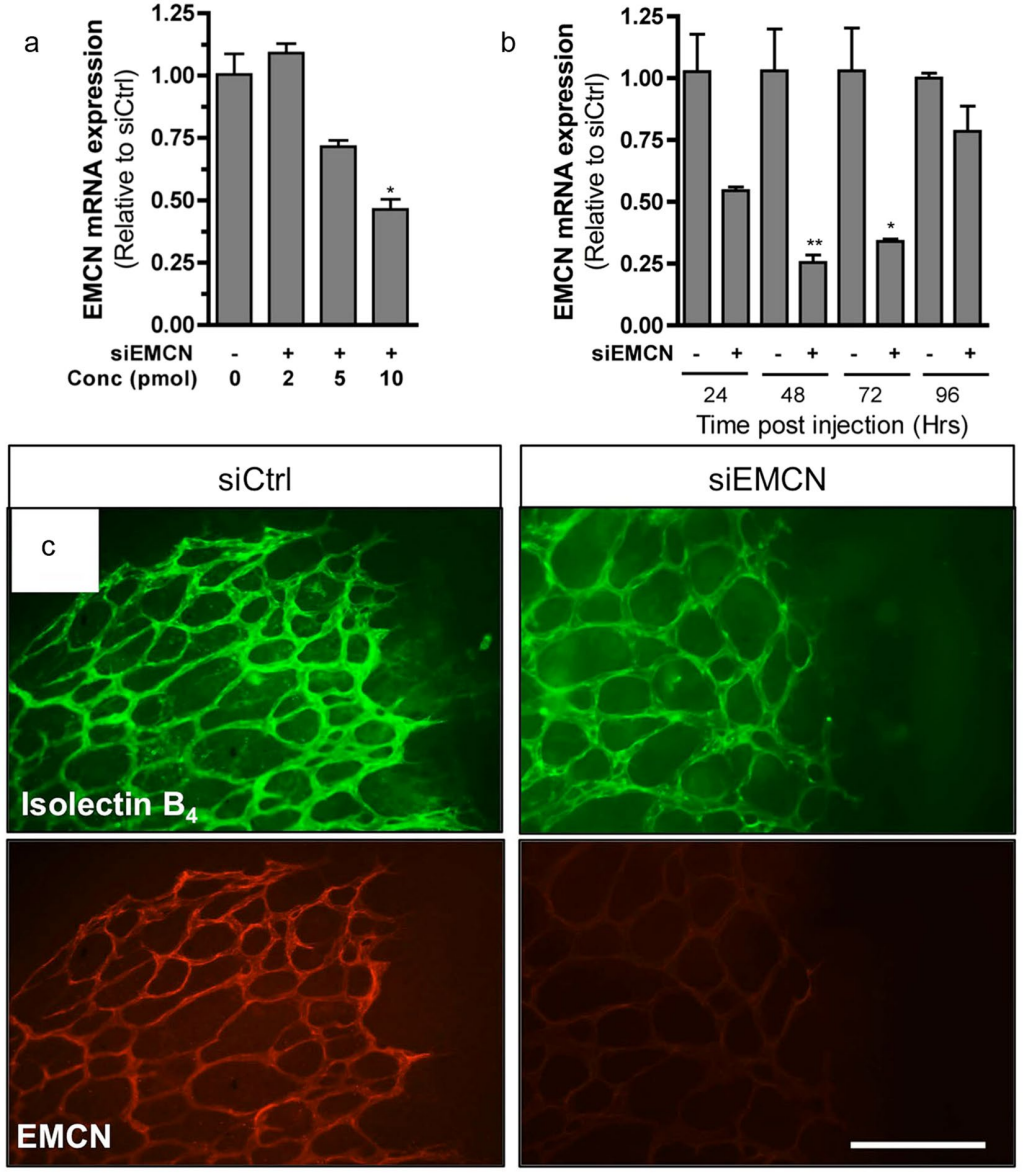
Inhibition of EMCN mRNA and protein levels *in vivo* after intravitreal injection. EMCN and control siRNA were injected directly into the vitreous humor of a P4 mouse eye and inhibition of EMCN levels were assessed by (**a**,**b**) qRT-PCR and (**c**) immunofluorescence. (**a**) Knockdown of EMCN mRNA levels was evaluated at 48 hr after injection using different concentrations of siEMCN compared to control. (**b**) Inhibition of EMCN mRNA was assessed at different time points post injection using 10 pmol of EMCN siRNA. (**c**) Flat-mounted images of isolectin B_4_ (green) and EMCN (red) stained retinas from P6 neonatal mice 48 hr post intravitreal injection of control or siEMCN (10 pmol) shows knockdown of EMCN expression. *P < 0.05, **P < 0.01, siEMCN vs siCtrl. Scale bar, 100 μm.

In light of its strong retinal vascular expression, we investigated the function of EMCN during developmental angiogenesis by analyzing the effects of EMCN knockdown during mouse neonatal retinal angiogenesis. Murine retinal vascularization is an ideal model to examine vascular development because it occurs postnatally and thus is easily accessible and observable, allowing for experimental manipulation^21^. Different concentrations of small interfering RNA (siRNA) against *Emcn* (siEMCN) were injected into the vitreous of P4 mouse eyes; the eyes were harvested two days later, and inhibition of EMCN mRNA was assessed by qRT-PCR and compared to non-targeting control siRNA (siCtrl) injected eyes. A comparison of a range of siEMCN concentrations (2–10 pmol) revealed 10 pmol as the minimal siEMCN dosage needed to suppress EMCN mRNA expression (Fig. 2a). In mice injected with 10 pmol siEMCN, EMCN mRNA levels were reduced within 24 hr and remained significantly reduced at 48 and 72 hr after injection compared to siCtrl-injected mice, and were nearly restored to control levels by 96 hr after injection (Fig. 2b). Whole mount retinas isolated 48 hr after intravitreal injection with 10 pmol of siEMCN and double-stained for isolectin-B_4_ (IB_4_) to label EC and EMCN revealed robust reduction of EMCN protein levels compared to siCtrl-injected mice (Fig. 2c). No alterations in IB_4_ and EMCN staining were observed in mice injected with the control *Trans*IT-TKO® transfection reagent (not shown).

**Figure 3:**
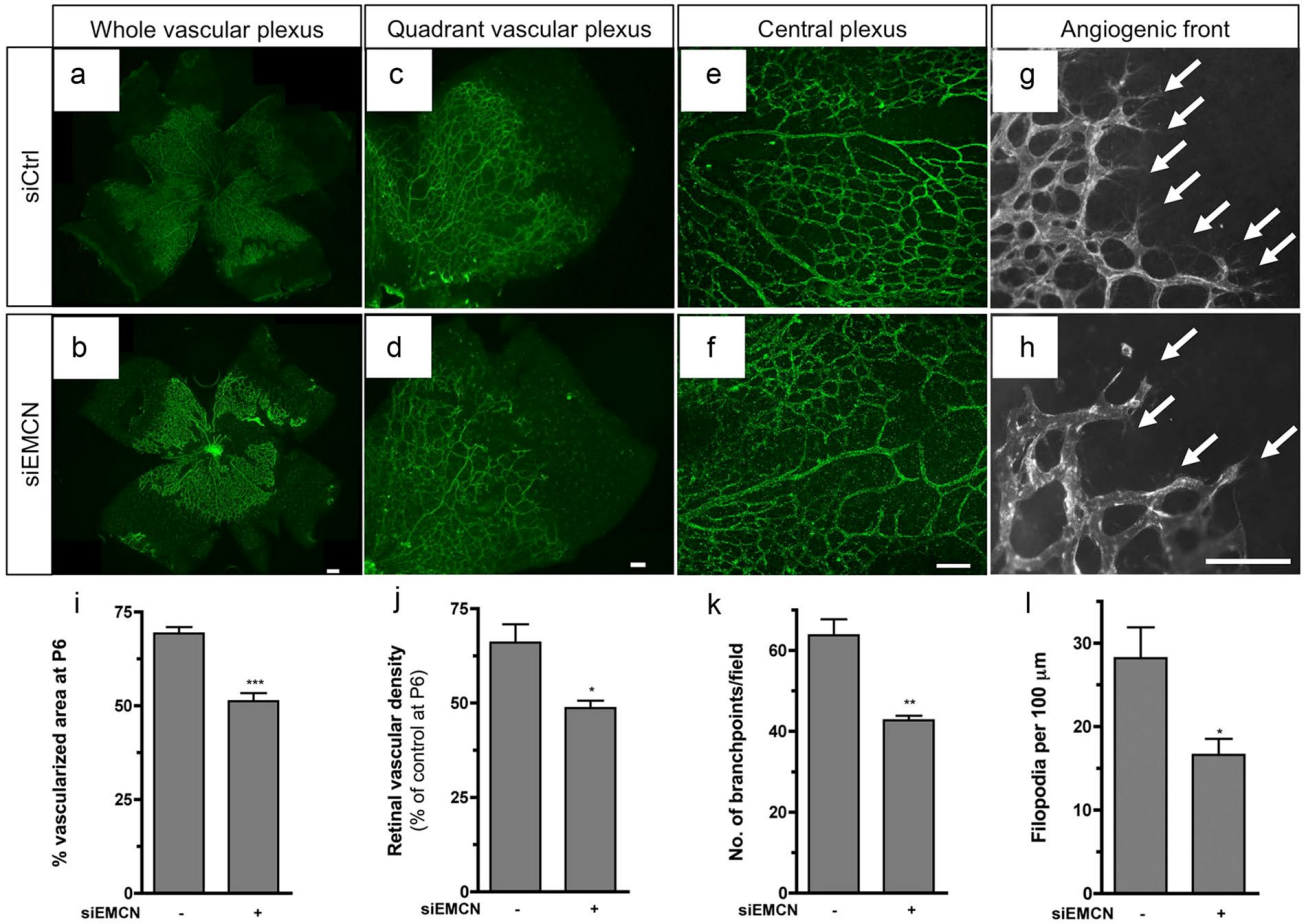
Loss of EMCN impairs angiogenic vessel growth. (**a**–**h**) Phenotype of isolectin B_4_-stained siCtrl and siEMCN retinal mouse vasculature. (**a**,**b**) Whole flat-mount retinas from P6 neonatal mice 48 hr post intravitreal injection. Higher magnifications of the (**c**,**d**) quadrant vascular plexus, (**e**,**f**) the central plexus, and (**g**,**h**) the angiogenic front are shown. (**i**) Vascular coverage and (**j**) density of siEMCN-injected mice show reduced vessel expansion to the periphery and reduced vessel density compared to siCtrl mice. (**k**) Branch points were counted in randomly selected 200 μm × 200 μm fields (five fields per retina; n = 10) of siEMCN and control retinas and (**l**) filopodia number was normalized to the outline of the angiogenic front. *P < 0.05, **P < 0.01, ***P < 0.001 siEMCN vs siCtrl. Scale bar, 100 μm.

Next, we evaluated the effect of EMCN knockdown on vascular development in P6 retinas. Mice injected with siEMCN showed a reduced development of the vascular plexus from the optic nerve head to the periphery compared to siCtrl-injected mice, indicative of impaired angiogenesis (compare Fig. 3a and b). Quantification of the developing vasculature confirmed a significant reduction in radial expansion in mice that had been injected with siEMCN (Fig. 3i). To quantify the effect of EMCN knockdown on angiogenesis, we examined vessel density, branch point number, and tip cell number in P6 retinas. All of these indexes were significantly decreased in siEMCN-injected mice compared with siRNA control injected littermate mice (Fig. 3e–h and j–l). Visualization of the forming vessel front at higher magnification revealed that the number of filopodia per 100 μm of leading endothelial membrane was reduced throughout the forming vascular networks (Fig. 3l), suggesting that the impaired vascularization with reduced EMCN might be attributable to decreased EC migration. There was no detectable difference in retinal EC proliferation as evidenced by immunostaining for phospho-histone H3 (PHH3), which labels mitotic cells, and revealed no significant difference in the number of proliferating cells between the siEMCN and siCtrl treated retina (Supplementary Fig. S1).

### EMCN regulates VEGF-induced EC migration

**Figure 4:**
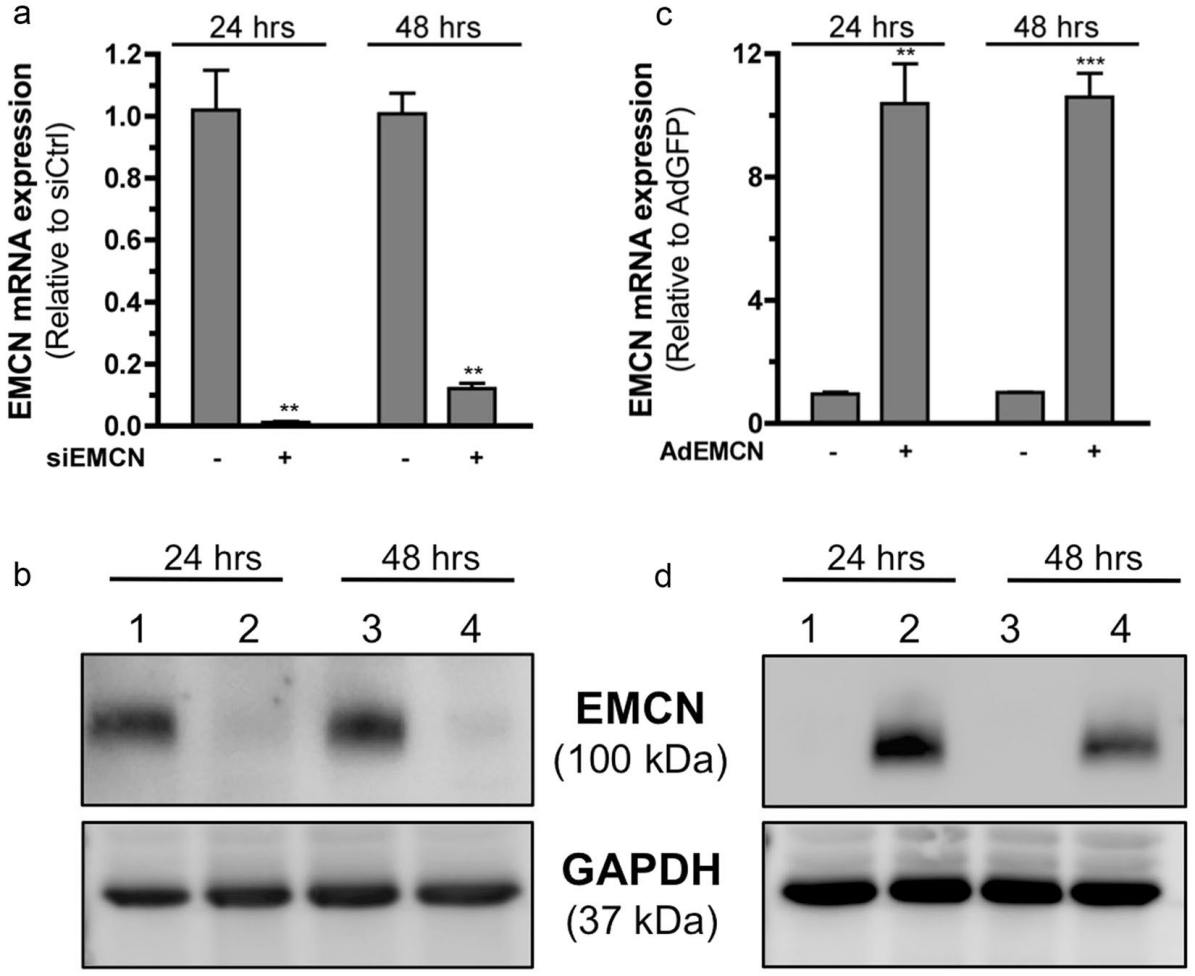
Knockdown and over-expression of EMCN on HRECs. (**a**,**b**) Sub-confluent HRECs were transfected with siRNA duplex directed against human EMCN (siEMCN), and control non-targeting siRNA (siCtrl). Effect of siEMCN compared to siCtrl were evaluated by (**a**) qRT-PCR and showed efficient reduction of EMCN mRNA levels at 24 and 48 hr respectively. (**b**) Western blot image of siCtrl (lanes 1, 3) and siEMCN (lanes 2, 4) at 24 and 48 hr after transfection were probed with antibodies recognizing human EMCN and GAPDH. (**c**,**d**) Sub-confluent HRECs were infected with adenoviruses expressing GFP (AdGFP) or EMCN (AdEMCN) at MOI 6 and protein biosynthesis of mouse EMCN and GFP was evaluated after 24 and 48 hr after infection. Over-expression of EMCN was assessed by (**c**) qRT-PCR and (**d**) western blot of AdGFP (lanes 1, 3) and AdEMCN (lanes 2, 4). **P < 0.01, ***P < 0.001 siEMCN vs siCtrl or AdEMCN vs AdGFP. Cropped gels are displayed.

In order to investigate the cellular basis of the effects of EMCN on angiogenesis, we performed a cell-based assay to examine the role of EMCN in EC migration. Treatment of human retinal endothelial cells (HREC) with siRNA sequences against *EMCN* (siEMCN) resulted in knockdown of *EMCN* expression by approximately 93% at 24 hr and about 85% after 48 hr, measured by qRT-PCR compared to siCtrl (Fig. 4a). We then evaluated EMCN protein expression in HRECs following treatment with siEMCN by immunoblot (Fig. 4b). A protein of 100 kDa, the predicted molecular weight of EMCN, was detected in the siCtrl-transfected cells (lanes 1 and 3 in Fig. 4b) whereas siEMCN treatment led to a 90% suppression of EMCN protein at 24 and 48 hr that was maintained up through 96 hr. To achieve over-expression, subconfluent HRECs were infected with adenovirus encoding EMCN (AdEMCN) or GFP-expressing adenovirus (AdGFP) control at a multiplicity of infection (MOI) 6. Exposure to AdEMCN resulted in a 10.6-fold increase in EMCN mRNA and an approximately 4-fold increase in protein (Fig. 4c and d) at 24 and 48 hr after infection.

**Figure 5:**
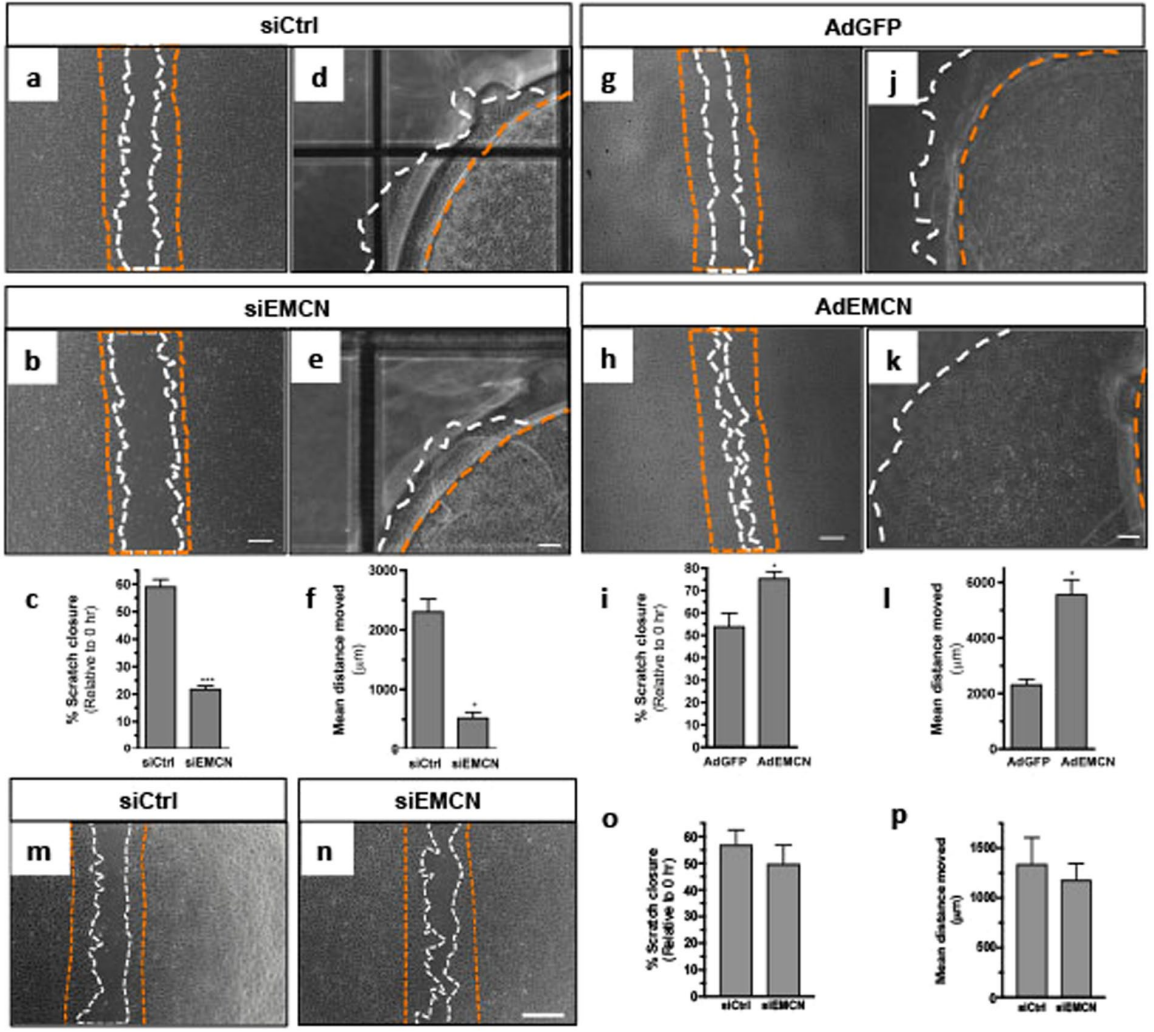
EMCN controls endothelial cell migration *in vitro*. HRECs were transfected with siCtrl, siEMCN, or infected with AdGFP or AdEMCN and cell migration was measured by a wound assay or an under-agarose assay. (**a**,**b**,**g**,**h**) Images of the wound margins immediately (orange) and 10 hr (white) after scratch in response to 10 ng/mL VEGF. (**d**,**e**,**j**,**k**) Images of cells contained in cut out wells (orange) migrating toward an adjacent well (white) containing 25 ng/mL VEGF at 36 hr. (**c**,**i**) Scratch closure was quantified at 10 hr and data are expressed as a % of the initial scratch width (**c**) for siCtrl and siEMCN or (**i**) AdGFP and AdEMCN treated cells. (**f**,**l**) Graph showing the mean distance moved of HRECs transfected with (**f**) siCtrl or siEMCN or infected with (**l**) AdGFP and AdEMCN at 36 hr. Data are expressed in μm and normalized to 0 hr. Scale bar 500 μm. (**m**,**n**) Representative images of the wound margins immediately (orange) and 10 hr (white) after scratch in response to 10 ng/mL bFGF. (**o**) Scratch closure was quantified at 10 hr after bFGF stimulation and data are expressed as a % of the initial scratch width. (**p**) Graph showing the mean distance moved of HRECs transfected with siCtrl or siEMCN at 36 hr after bFGF treatment. Results are from four independent experiments in triplicate. *P < 0.05, ***P < 0.001 siEMCN vs siCtrl or AdEMCN vs AdGFP. Error bars represent SEM.

The effect of EMCN reduction on the migration of HRECs was examined using an established *in vitro* EC wound-healing assay. Cells with reduced levels of EMCN exhibited reduced migration into the wounded area in response to VEGF compared to the control (Fig. 5a and b). Data collected from three separate experiments confirmed that cells with reduced EMCN had less wound-closure (67% less than controls) at 10 hr post-wounding (Fig. 5c). Similar results were observed using human umbilical vein endothelial cells (Supplementary Fig. S2). The effect of EMCN over-expression on migration in response to VEGF compared to control GFP over-expressing cells was tested. In contrast to the effects of EMCN knockdown, AdEMCN infected cells exhibited a 1.4-fold increase in migration at 10 hr post-wound when compared with AdGFP control infected cells (Fig. 5g–i).

Given that reduced levels of EMCN impaired cell movement in a wound assay, we utilized a chemotaxis assay to determine if EMCN knockdown affected directed EC motility. To accomplish this, we used an under-agarose assay, which involves creating wells in agarose and adding the chemoattractant to one well and HRECs to the second well. Directed cell migration was documented at various time points over 36 hr. Comparison of the effects of 10 and 25 ng/mL of VEGF in an EC under-agarose migration assay established 25 ng/mL as the optimal VEGF dosage for the stimulation of HREC migration. HRECs in which EMCN was knocked down with siEMCN migrated up to 0.5 mm from the starting point whereas control cells (treated with siCtrl) migrated up to 2.3 mm (Fig. 5d–f). The migration of AdEMCN infected cells in response to VEGF at 36 hr was increased 2.6-fold compared to that of AdGFP-infected control cells (Fig. 5j–l). In addition, while it appears that angiogenesis in a majority of situations is mediated by VEGF, bFGF is also well known as a potent angiogenic factor. We therefore determined whether EMCN regulation is altered during migration following stimulation with bFGF. Using both the wound-healing assay and the chemotaxis assay, cells with reduced levels of EMCN did not exhibit a significant change in migration in response to bFGF compared to the control (Fig. 5m–p).

### EMCN regulates VEGF-stimulated EC proliferation

**Figure 6:**
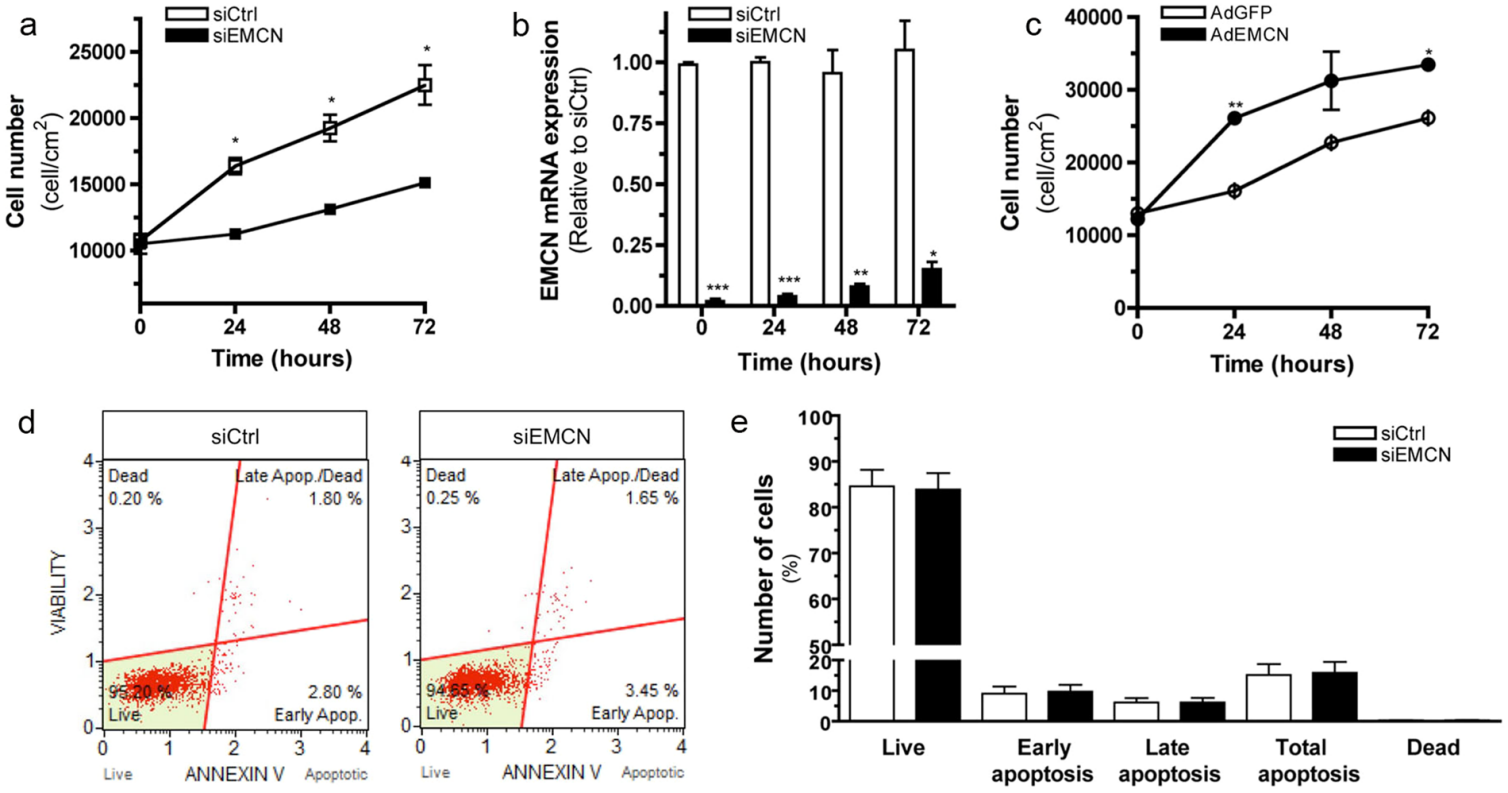
EMCN regulates HREC proliferation. Cell growth and viability as measured by (**a**,**c**) Trypan blue or assessed for cell death using a (**d**,**e**) Muse automated cell analyzer. (**a**) Graph showing cell number of siEMCN (closed black squares) and siCtrl (open squares) transfected cells 0, 24, 48, and 72 hr after treatment with VEGF (10 ng/mL) with (**b**) corresponding mRNA levels of EMCN at the time points indicated shows significant reduction in EC proliferation due to loss of EMCN expression. (**c**) Graph showing cell number of AdEMCN (closed black circles) and AdGFP (open circles) infected cells 0, 24, 48, and 72 hr after treatment with VEGF (10 ng/mL) shows enhanced EC cell growth. (**d**,**e**) After 24 hr transfection with siEMCN or siCtrl, induction of apoptosis was measured using a Muse automated cell analyzer. (**d**) Plots of three independent siEMCN and siCtrl experiments performed for annexin V detection shows non-apoptotic live (lower left: 7-AAD negative, apoptosis negative), non-apoptotic dead (upper left: 7-AAD positive, apoptosis negative), apoptotic live (lower right: 7-AAD negative, apoptosis positive), and apoptotic dead (upper right: 7-AAD positive, apoptosis positive) cells. (**e**) Percentage of cells in five populations identified with annexin V is shown. *P < 0.05, **P < 0.01, ***P < 0.001 siEMCN vs siCtrl or AdEMCN vs AdGFP.

We next determined whether EMCN influences EC viability and growth in response to growth factor withdrawal and VEGF exposure. Cell viability and growth were measured using the trypan blue exclusion assay on cells with EMCN knockdown or control cells. Cells were cultured for 0, 24, 48, and 72 hr in serum-free medium with and without VEGF. In the presence of VEGF (10 ng/mL), EMCN-deficient cells displayed a significant decrease in cell proliferation (50% decrease) compared to control cells (Fig. 6a). Reduced EMCN mRNA expression was confirmed at each time point (Fig. 6b).

To assess whether reduction in cell proliferation by EMCN knockdown was due to reduced proliferation or increased cell death, the percentage of apoptotic cells was determined using a Muse automated cell analyzer 48 hr after siEMCN transfection and cultured in VEGF- supplemented serum-free medium. Cytofluorimetric analysis of annexin V positive HRECs showed no change in the percentage of apoptotic cells upon siEMCN treatment compared with the siCtrl control (Fig. 6d and e). Similar results were obtained using HUVEC (Supplementary Fig. S2). Thus, reduced cell number of siEMCN transfected cells was due to diminished proliferation and not cell death. In contrast, EMCN over-expression led to significant increase in VEGF-induced cell proliferation compared to control cells over-expressing GFP (Fig. 6c).

### EMCN expression modulates VEGF-induced tube morphogenesis by HRECs

**Figure 7:**
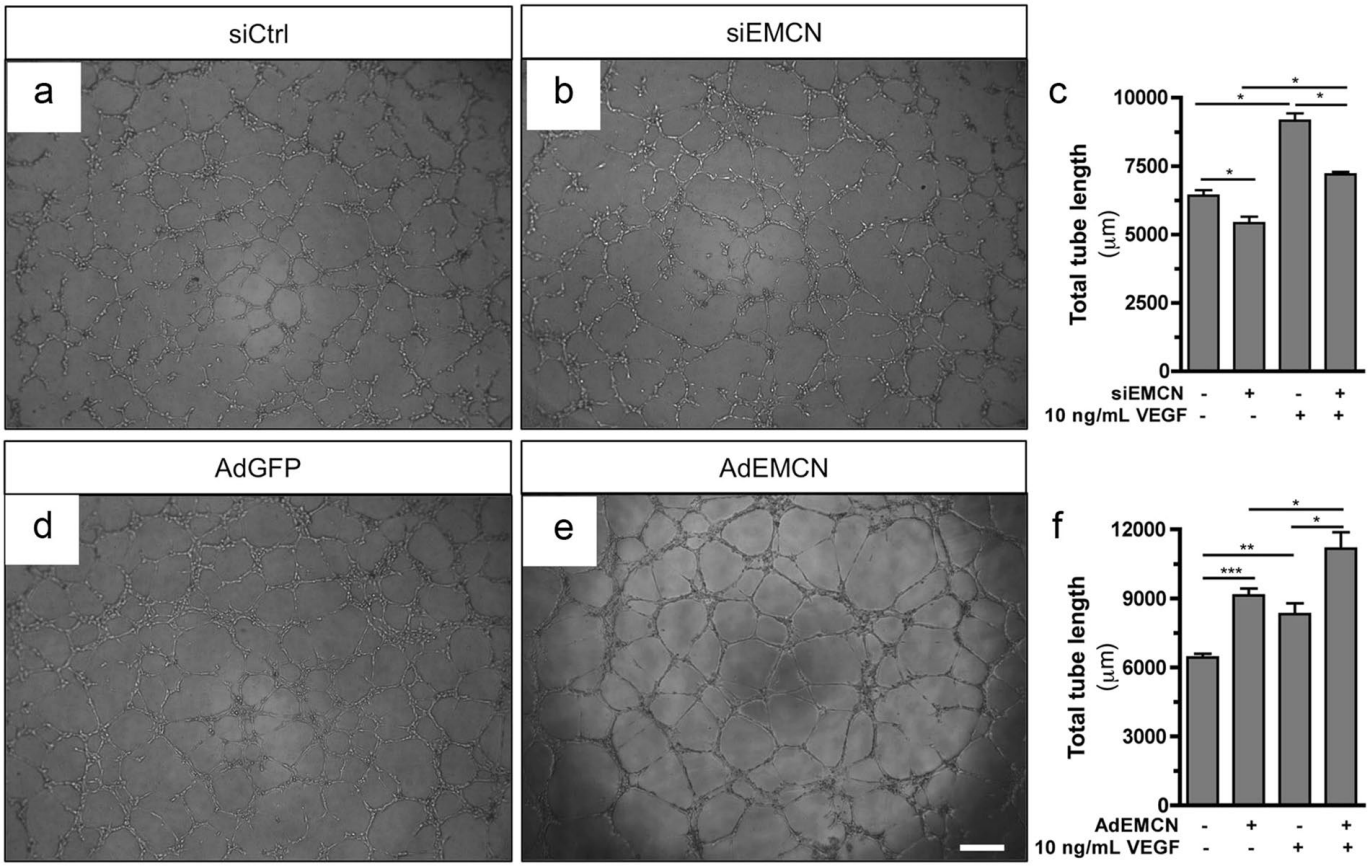
EMCN expression modulates ECM-induced tube morphogenesis. Representative 6 hr images of HRECs transfected for 24 hr with (**a**) siCtrl or (**b**) siEMCN or infected with (**d**) AdGFP or (**e**) AdEMCN plated on polymerized basement membrane extract (BME) in the presence of VEGF (10 ng/mL) are shown. (**c**,**f**) Quantification of total tube length 12 hr after plating on BME in the presence and absence of VEGF shows (**c**) knockdown of EMCN expression inhibits ECM-induced tube formation while (**f**) over-expression of EMCN enhances ECM-induced tube formation. *P < 0.05, ***P < 0.001 siEMCN vs siCtrl or AdEMCN vs AdGFP; *P < 0.05, **P < 0.01 VEGF vs no VEGF. Scale bar 100 μm.

To investigate the involvement of EMCN endothelial morphogenesis, we employed an *in vitro* model of tube formation in which ECs assemble into vessel-like tubes containing lumens. To assess a possible role for EMCN in tube formation, ECs were transfected with siEMCN or siCtrl for 24 hr and an image of the central area of each well was captured at the 0 time point and the same field was monitored until the peak of tube formation or until the tubes had disassembled. The data revealed that tube formation by EC with reduced EMCN expression was significantly inhibited (Fig. 7a and b). Unlike the cells treated with siCtrl, a majority of the cells with reduced EMCN expression displayed a rounded morphology and did not organize into tube-like structures. In addition to the tube formation assay, a 3-dimensional spheroid assay was also carried out with similar results (Supplementary Fig. S3).

Results demonstrated that tube formation by EC with reduced EMCN was inhibited by approximately 22% and 18% in the absence or presence of VEGF after 12 hr, respectively (Fig. 7c). The number of tubes formed by EMCN-reduced cells was significantly less than that formed in the control culture at later time points (18–24 hr after cell seeding) (Supplementary Fig. S4). Conversely, the morphogenic response of AdEMCN infected cells was increased by 1.4- and 1.3-fold over that observed in AdGFP infected control cells in the absence or presence of VEGF at 12 hr, respectively (Fig. 7d–f).

### EMCN controls angiogenesis by altering VEGFR2 activation

**Figure 8:**
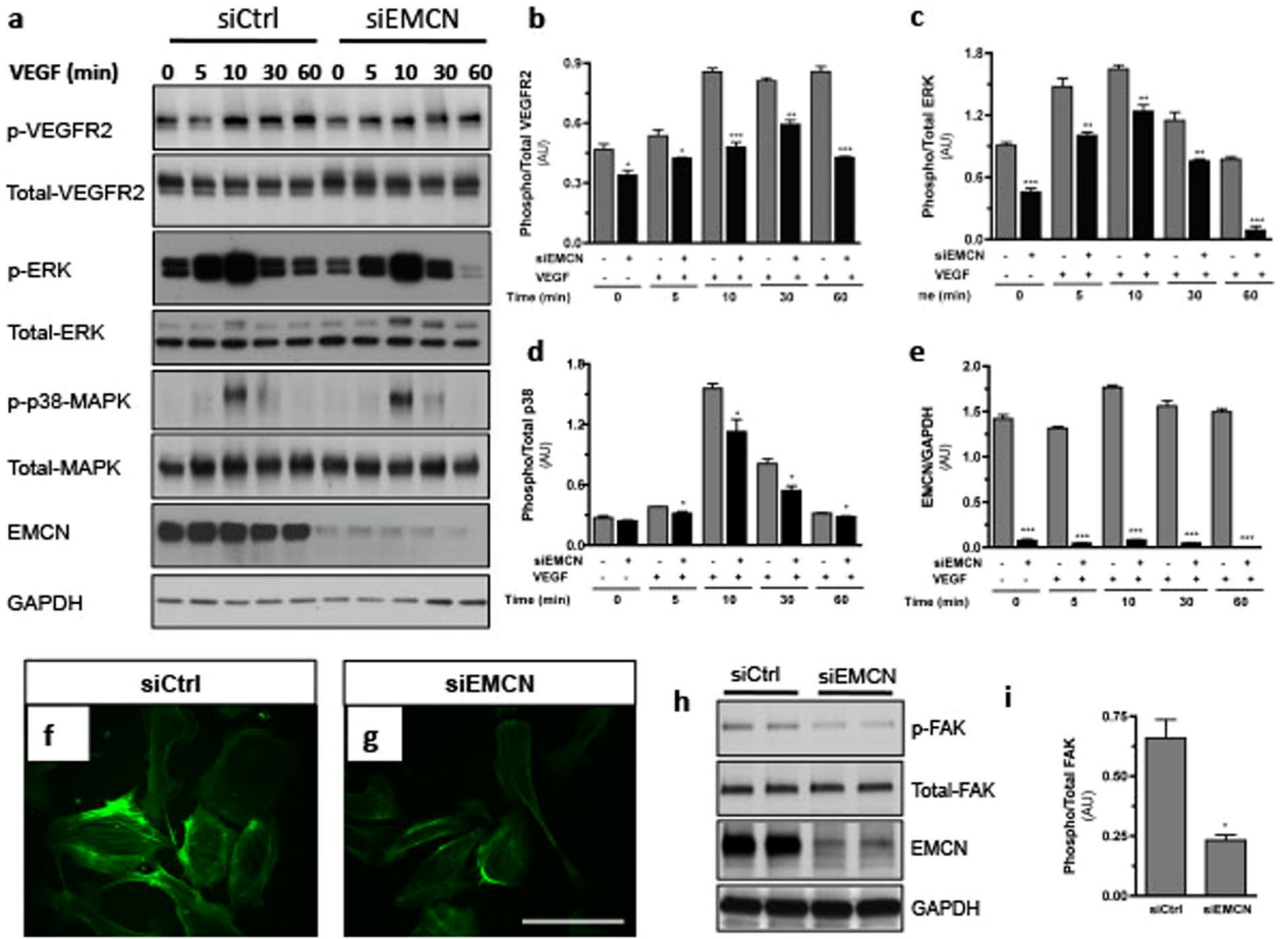
EMCN-knockdown is associated with altered VEGFR2 signaling. (**a**) Immunoblot and (**b**–**e**) quantitative analysis of siCtrl and siEMCN cells stimulated with exogenous VEGF (10 ng/mL) at indicated time points assessed for levels of phosphorylated and total VEGFR2, ERK1/2, p38-MAPK, EMCN and GAPDH. (**f**,**g**) Rhodamine-phalloidin staining of HRECs shows that silencing EMCN dramatically inhibits the formation of actin stress-fibers. (**h**,**i**) Immunoblot and quantitative analysis shows reduction of phospho-FAK in siEMCN treated cells. Results are from four independent experiments in triplicate. *P < 0.05, **P < 0.01, ***P < 0.001. Error bars represent SEM. Scale bar 100 μm. Cropped gels are displayed.

VEGFR2 is the key signaling receptor for VEGF and has been shown to mediate angiogenesis and capillary EC migration^22,23^. To gain insight into the mechanism by which EMCN influences angiogenesis we investigated whether EMCN is involved in VEGF-VEGFR2 signaling. HRECs with or without siEMCN-mediated knockdown were stimulated with exogenous VEGF (10 ng/mL) for different times and assessed for levels of VEGFR2 phosphorylation by immunoblot (Fig. 8a). VEGF treatment of cells with reduced EMCN displayed an approximately 45% reduction in VEGFR2 phosphorylation compared to siCtrl cells (Fig. 8b). The basal level of phospho-VEGFR2 was also significantly reduced in siEMCN treated cells compared to siCtrl cells in the absence of VEGF treatment. ERK1/2 and p38-MAPK are known downstream components of VEGFR2 signaling^24^. Examination of the effect of EMCN knockdown on the activation of ERK1/2 and p38-MAPK revealed a decrease in phospho-ERK1/2 and phospho-p38-MAPK levels in cells with reduced EMCN in response to VEGF treatment (Fig. 8c and d). Of note, we also observed a significant decrease in phospho-Akt levels in response to VEGF treatment (Supplementary Fig. S5). These findings clearly indicate that EMCN plays a role in modulating VEGF-induced VEGFR2 activation.

The activation of VEGFR2 by VEGF in ECs is associated with the remodeling of the actin cytoskeleton. Thus, the effect of EMCN knockdown on the actin cytoskeleton was evaluated by rhodamine-phalloidin staining of HRECs 48 hr after transfection. siEMCN transfected cells had significantly fewer actin filaments compared with the siCtrl transfected cells (Fig. 8f and g). Consistent with the loss of actin stress-fibers, suppression of EMCN expression in HRECs also led to a decrease in the expression of focal adhesion kinase (FAK), which is found at the cell membrane where the cytoskeleton interacts with proteins of the extracellular matrix. EMCN knockdown also downregulated tyrosine phosphorylation of FAK protein (Fig. 8h and i), suggesting a dependence on FAK signaling.

## Discussion

The endothelial glycocalyx is a collection of proteoglycans and glycoproteins on the apical endothelial surface. Among other roles, the glycocalyx appears to contribute to the maintenance of the vessel wall^25^. Under normal conditions, the endothelial glycocalyx has been shown to be an important determinant of vascular permeability^26^, to attenuate blood cell–vessel wall interactions^27^, to function as a mechanosensor^28^, to enable specific cell signaling processes (e.g. cytoskeletal reorganization)^29^, and to fulfill a vasculoprotective role^30^. Loss or disruption of the glycocalyx can contribute to the development of pathology.

EMCN is a relatively understudied, but major component of the endothelial glycocalyx. It has become useful as a marker of capillaries, in general^15^, and tumor capillaries, in particular^16^. In light of our observation that EMCN loss was associated with impaired angiogenesis in cystic embryoid bodies derived from VEGF-null embryonic stem cells and with increasing evidence pointing to a role for components of the glycocalyx in vessel formation^19^, we tested whether EMCN, a mucin-like glycoprotein, contributes to the regulation of vascular stability and angiogenesis. Our results indicate that EMCN impacts angiogenesis by modulating the VEGF pathway via its primary signaling receptor VEGFR2.

A role for mucins in angiogenesis is not without precedent. Studies of mucin 1 (MUC1), a membrane-bound glycoprotein that is expressed by various epithelial cell types, have shown that upregulation of MUC1 favors tumor angiogenesis in non-small-cell lung cancer, likely through the activation of both Akt and ERK1/2 pathways and elevation of VEGF production^31^, and that MUC1 enhances hypoxia-driven angiogenesis^32^.

Angiogenesis is a complex process comprised of proteolytic degradation of the matrix followed by EC migration, proliferation and morphogenesis^4^. VEGF has been shown to play an important role in angiogenesis and cell survival pathways, and has been demonstrated to regulate the cytoskeleton, as well as other associated proteins^33,34^. Members of the VEGF family bind to receptor tyrosine kinases such as VEGFR1 (Flt-1)^35^, VEGFR2 (KDR or FIk-1)^36^, and VEGFR3 (FIt-3)^37^ and co-receptors heparan sulfate proteoglycans (HSPGs) and neuropilins (NRP). VEGFR2 appears to be the most important receptor in VEGF-induced angiogenesis and has been shown to be involved in all aspects of normal and pathological angiogenesis. VEGF binding to VEGFR2 leads to phosphorylation of Tyr1175 and initiates a signaling cascade leading to angiogenesis, permeability or survival^38^. Tyrosine phosphorylation of VEGFR2 is inhibited by the activation of protein tyrosine phosphatases (PTPs), including Src homology- 2-domain-containing protein tyrosine phosphatase 2 (SHP-2)^39^ and PTP1B^40^. VEGFR2 autophosphorylation is required for activation of diverse signaling pathways such as ERK1/2 and p38-MAPK, which are involved in EC proliferation and migration, respectively^10,11^. We demonstrate that EMCN knockdown led to reduced VEGFR2 phosphorylation and significant suppression of ERK1/2 and p38-MAPK activation, known components of VEGF downstream signaling.

We speculate that EMCN may impact VEGFR2 phosphorylation levels by influencing VEGF binding to VEGFR2. In support of this possibility, the extracellular domain of EMCN includes a potential HSPG-binding domain and VEGFR2 signaling has been reported to be modulated by interaction with cell surface HSPG^41^. Furthermore, endothelial HSPGs modulate the interaction of heparin-binding VEGFs with signaling VEGFRs and NRP co-receptors^42^. Recently, it was reported that HSPGs on the cell surface of ECs may function as a common modulator of VEGF binding to various receptors/co-receptors, and may participate in interactions of these receptors/co-receptors with one another^43^. Alternatively, EMCN may interact with other carbohydrate-binding proteins, such as the different galectins, via its extensive O-glycosylation and indirectly modulate VEGFR2 function in angiogenesis. The critical role of the various galectins in angiogenesis has been established. For example, galectins -1, -3, -8 and -9 have all been shown to influence angiogenic processes by engaging a different set of EC surface receptors, activating distinct signaling pathways thereby regulating different events in the angiogenic cascade^44^.

Migration of ECs is central to the process of vessel morphogenesis^23^ and it is well known that the cytoskeleton is integral to the regulation of cellular morphology and movement. ECs with reduced EMCN *in vivo* extended significantly fewer lamellipodia compared to the EC in control eyes. The formation and extension of lamellipodia have been clearly demonstrated to require actin polymerization and to involve Rac and Arp2/3 complex, and induction of actin filament via VASP^45^. Actin polymerization is required for the formation of F-actin, structurally important for actin-based cytoskeletal structures and stress fibers. In cultured ECs with reduced EMCN expression, we observed decreased F-actin expression, as visualized by immunofluorescence, indicating that the presence of EMCN is necessary for actin polymerization in ECs. Members of the Rho family of small GTPases regulate the actin cytoskeleton, cell polarity, and microtubule dynamics and are also downstream targets of VEGFR2. The link between VEGFR2 and RhoA further involves activation of the Rho-associated kinase (ROCK) and FAK. Rho/ROCK signaling regulates VEGF-mediated migration, cell permeability and survival^46^, while FAK integrates the signal between VEGFR2 and integrin αvβ3 and controls the assembly and disassembly of focal adhesions^47^. We found that ECs with reduced EMCN resulted in reduced phosphorylation of VEGFR2 as well as FAK formation, suggesting that EMCN regulates EC migration by modulating FAK formation. Furthermore, activation of PI3K, an important determinant of EC motility^48^, also influences cell proliferation via the generation of phosphoinositides. In particular, PI3K contributes to the activation of 3-phosphoinositide-dependent protein kinase-1 (PDK1), which leads to the activation of Akt/PKB^49^, and consistent with a role of EMCN in EC motility that VEGF-mediated activation of Akt/PKB was also greatly reduced upon EMCN knockdown in ECs.

Our results demonstrate that EMCN plays a central role in normal angiogenesis, revealing a novel function for EMCN in regulation of proangiogenic signaling in EC migration, proliferation, and morphogenesis. VEGFR2 has been shown to be expressed by a wide variety of non-endothelial cells including ependymal cells that line the brain ventricles^50^ and retinal pigment epithelial cells^51^, where it acts as a survival factor, as well as by photoreceptors^52^ and retinal ganglion cells^53^, where is serves a neuroprotective role. Because of EMCN’s specific expression by the endothelium, blocking VEGF signaling by targeting EMCN would provide a specific and attractive therapeutic target for treatment of angiogenesis-related diseases.

## Methods

### Ethics Statement

The Harvard Medical Area Standing Committee on Animals approved all animal procedures. Mice were handled in accordance to the National Institute of Health Guide for the Care and Use of Laboratory Animals. All procedures were approved by the Institutional Animal Care and Use Committee of the Schepens Eye Research Institute/Mass Eye and Ear.

### Mice

C57BL/6J mice were purchased from Jackson Laboratories.

### Reagents and antibodies

Non-targeting control siRNA (siCtrl) and siRNA directed against EMCN (siEMCN; Dharmacon) were purchased as SMART pools. Dharmafect 1 transfection reagent (Dharmacon) was purchased for cell culture studies while *in vivo* studies used transit-TKO transfection reagent (Mirius Bio). A cDNA encoding full-length murine *Emcn* (provided by Dietmar Vestweber, Max-Planck-Institute, Germany) was cloned into a pShuttle vector and enhanced green fluorescent protein (EGFP) was cloned into pAdEasy. Adenoviral vectors were amplified in DH5alpha cells and purified plasmids were linearized and transfected into 293A cells, tittered by optical absorbance method, and expressed as plaque forming units (pfu) per ml. The EMCN adenovirus titer is 3.1 × 10^10^ pfu/mL and EGFP adenovirus titer is 1 × 10^11^ pfu/mL. The virus is distributed in the following formulation: 50 mM Tris-HCl, pH 7.4, 5 mM EDTA, 1.4 M CsCl, 50 mM NaCl, 0.5 mM MgCl2, and 25% glycerol. Retinas were stained with Alexa 488-labeled isolectin-B_4_ (Life Technologies), rat anti-EMCN (Santa Cruz Biotechnology), and rabbit anti-phospho-histone H3 (Millipore) while immunoblots were probed with antibodies against EMCN (Abcam), rabbit anti-GAPDH (Santa Cruz Biotechnology), phospho-Y951-VEGFR2, VEGFR2, phospho-p38-MAPK, p38-MAPK, phospho-ERK, ERK, phospho-FAK, and FAK (Cell Signaling).

### Retinal vessel development *in vivo*

siRNAs were injected intravitreally under a dissecting microscope into P4 C57BL/6 J mice using a Hamilton syringe with a 33-gauge blunt-ended needle. Retinas were incubated overnight at 4 °C with the appropriate antibodies and analyzed with an Axioskop 2 Mot Plus microscope (Carl Zeiss).

### Cell culture

Human retinal endothelial cells (HRECs; Cell Systems) were cultured on 0.2% gelatin-coated dishes in EGM-2 Bulletkit medium (Lonza) supplemented with 2% fetal bovine serum (Atlanta Biologicals), 2 mM L-glutamine (Lonza), and 100 U/mL penicillin/100 μg/mL streptomycin (Lonza). Cells were maintained at 37 °C with 5% CO2. HRECs were used within passages 6–10.

#### siRNA knockdown

At 60% confluence, siEMCN or siCtrl were incubated with Dharmafect 1 transfection reagent at room temperature for 20 min to form complexes, and added to cells at a final siRNA concentration of 200 pM.

#### Adenovirus infection

HRECs were seeded at 60% confluence one day prior to adenoviral infection. Cells were infected with AdGFP or AdEMCN at a multiplicity of infection 6.

### Migration assays

#### Wound-healing

Confluent monolayer of siRNA transfected or adenovirally infected HRECs (after overnight serum-starvation) were mechanically scratched using a P200 pipette tip. Cells were washed and replaced with the desired medium. An image of the same field was acquired over time and wound closure was quantified by the percentage change in the wound area per unit time.

#### Under-agarose

Two-mm wells were cut out of hardened agarose with VEGF (25 ng/mL) added to one well and siRNA transfected or adenovirally infected HRECs plated in a nearby well. Distance between the two wells was approximately 10 mm apart. Migration towards VEGF was monitored from time 0 up to 48 hr.

### Trypan blue exclusion assay

HRECs were plated at a density of 2.0 × 10^4^ cells per well of a 24-well plate and transfected with siRNA or infected with adenovirus for 24 hr. At different time points after transfection, the cell suspension was mixed with 0.02% trypan blue and incubated at room temperature for 3 min. Viable and nonviable cells were counted using a hemocytometer.

### Annexin-V staining by Muse cytofluorimetric analysis

HRECs were seeded into 12-well plates and transfected with siRNA or infected with adenovirus. The number of apoptotic cells was determined using the MUSE Annexin V and Dead Cell Kit (Millipore), according to the manufacturer’s instructions.

### Tube formation assay

Eighty microliters of basement membrane extract (BME; Trevigen) was polymerized in a 96-well plate at 37 °C for 30 min prior to seeding 1.8 × 10^4^ transfected or infected HRECs on top of BME. Culture medium with VEGF (10 ng/mL) was added to induce tube formation. The central area of each well was monitored over time where the total length of all tubes within a field was measured.

### Spheroid sprouting assay

HRECs were transfected with siCtrl or siEMCN overnight and were suspended and aggregated overnight to form cellular spheroids (500 cells/spheroid). HREC spheroids were embedded into collagen gels treated for 12 hr with 5 ng/ml VEGF. Spheroids were imaged at 4, 8, 12, and 24 hr.

### Analysis of protein expression

Cells were lysed with buffer containing protease inhibitor (Roche) and a phosphatase inhibitor cocktail tablet (Sigma). Protein concentration was determined using the BCA Assay (Thermo Scientific). Proteins separated on SDS-PAGE were transferred to PVDF membranes (Millipore) and then probed with appropriate antibodies. Proteins of interest were visualized by chemiluminescence.

### Analysis of mRNA expression

Total RNA was isolated from tissue and cells using RNA-Bee reagent (Amsbio) and was reverse transcribed into cDNA using iSCRIPT (BioRad). Reactions were performed on the LightCycler 480 II (Roche) using 0.4 uM primers and Faststart Universal SYBR Green PCR Master Mix (Applied Biosystems). Primer sequences are described in Supplementary Table S1.

### Image processing

ImageJ (National Institute of Health) and Photoshop CS6 (Adobe) software were used for image processing.

### Statistical Analysis

All values are expressed as mean ± SEM (unless specified). Statistical analysis was performed using an unpaired Student t test (GraphPad Prism 5). A *P* value < 0.05 was considered statistically significant. Each experimental condition was evaluated in triplicate or quadruplicate, and all experiments were independently repeated at least three times.

### Electronic supplementary material

Supplementary Information
