# Author Correction: Endomucin knockdown inhibits VEGF-induced endothelial cell migration, growth, and morphogenesis by modulating VEGFR2 signaling

**DOI:** 10.1038/s41598-023-43612-x

**Published:** 2023-10-03

**Authors:** Cindy Park-Windhol, Yin Shan Ng, Jinling Yang, Vincent Primo, Magali Saint-Geniez, Patricia A. D’Amore

**Affiliations:** 1 Schepens Eye Research Institute/Massachusetts Eye and Ear, Boston, MA USA; 2 Department of Ophthalmology, Harvard Medical School, Boston, MA USA; 3 Department of Pathology, Harvard Medical School, Boston, MA USA

Correction to: *Scientific Reports* 10.1038/s41598-017-16852-x, published online 07 December 2017

This Article contains an error in the Title,

“Endomucin inhibits VEGF-induced endothelial cell migration, growth, and morphogenesis by modulating VEGFR2 signaling.”

should read:

“Endomucin knockdown inhibits VEGF-induced endothelial cell migration, growth, and morphogenesis by modulating VEGFR2 signaling.”

