# Supplementary material for: Endomucin inhibits VEGF-induced endothelial cell migration, growth, and morphogenesis by modulating VEGFR2 signaling
Source: Sci Rep. 2017 Dec 7;7:17138. doi: 10.1038/s41598-017-16852-x (PMC5719432; doi:10.1038/s41598-017-16852-x)
Supplement: Supplementary file 1 — Supplementary Information [file 41598_2017_16852_MOESM1_ESM.pdf]

# Endomucin inhibits VEGF-induced endothelial cell migration, growth, and morphogenesis by modulating VEGFR2 signaling

Cindy Park-Windhol, PhD[1,2]; Yin-Shan Ng[1,2]; PhD; Jinling Yang, PhD[1,2]; Vincent Primo, BS[1,2]; Magali Saint-Geniez, PhD[1,2,*]; Patricia A. D'Amore, PhD[1,2,3,*]

[1]*Schepens Eye Research Institute/Massachusetts Eye and Ear, Boston, MA, USA*

[2]*Departments of Ophthalmology and* [3]*Pathology, Harvard Medical School, Boston, MA, USA*

**Magali Saint-Geniez, PhD and Patricia A. D'Amore, PhD are co-senior authors on this manuscript.*

**SUPPLEMENTAL FIGURES**

**Table S1: Sequences for primers for qRT-PCR.**

| | Forward | Reverse |
|---|---|---|
| h*EMCN*: | 5’TGCAGGACTTTCTCCTTTTC3’ | 5’ATTTGTTCTGGTGGGTTTGT3’ |
| h*GAPDH* | 5'CAAATTCCATGGCACCGTCA3' | 5'GGAGTGGGTGTCGCTGTTGA3’ |
| h*HPRT1* | 5'CCTGGCGTCGTGATTAGTGAT3' | 5'AGACGTTCAGTCCTGTCCATAA3' |
| m*Emcn* | 5'AATACCAGGCATCGTGTCAGT3' | 5'CTGATTCTCAGTCTTGTTCTGGG3' |
| m*Gapdh* | 5'GTGGCAAAGTGGAGATTGTTGCC3' | 5'GATGATGACCCGTTTGGCTCC3' |
| m*Hprt1* | 5'TCAGTCAACGGGGGACATAAA3’ | 5'GGGGCTGTACTGCTTAACCAG3' |

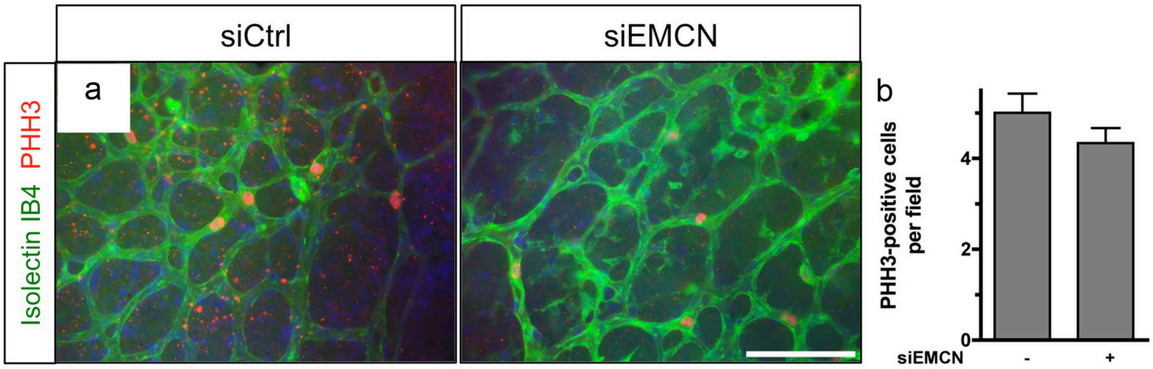


**<u>Figure S1</u>: Loss of EMCN shows a reduced apoptotic profile independent of proliferation.** **(a)** Representative images of a P6 vasculature labeled for $IB_4$ (green) and mitosis (phospho-histone; PHH3, red) and nuclei (blue) 48 hr after intravitreal injection with siEMCN or siCtrl. **(b)** Quantification shows no change in the number of PHH3-positive cells in siEMCN injected eyes compared to control eyes. Scale bar, 100 µm.

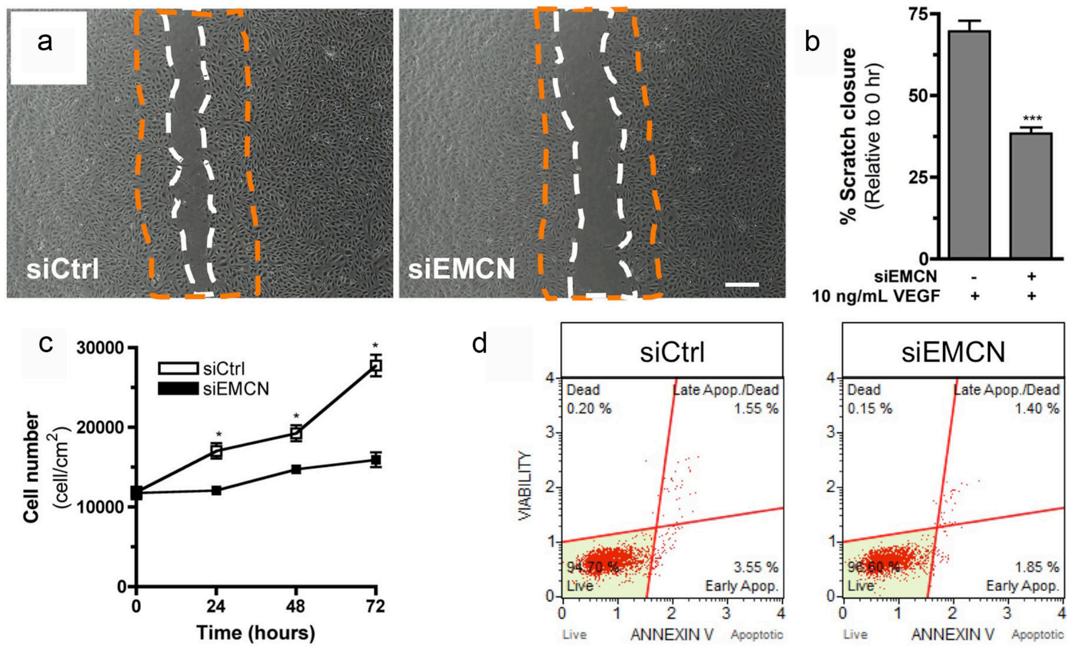


**<u>Figure S2</u>: EMCN controls EC migration and proliferation in HUVECs.** HUVECs were transfected with siCtrl or siEMCN. **(a, b)** Cell migration was measured by a wound assay and cell growth and viability were measured by **(c)** Trypan blue or assessed for cell death using a **(d)** Muse automated cell analyzer. **(a)** Representative images of the wound margins immediately (orange) and 10 hr (white) after scratch in response to 10 ng/mL VEGF. **(b)** Scratch closure was quantified at 10 hr and data are expressed as a % of the initial scratch width. **(c)** Graph showing cell number of siEMCN (closed black squares) and siCtrl (open squares) transfected cells 0, 24, 48, and 72 hr after treatment with VEGF (10 ng/mL) shows significant reduction in EC proliferation. **(d)** Representative plots of siEMCN and siCtrl experiments performed for annexin V detection shows non-apoptotic live (lower left: 7-AAD negative, apoptosis negative), non-apoptotic dead (upper left: 7-AAD positive, apoptosis negative), apoptotic live (lower right: 7-AAD negative, apoptosis positive), and apoptotic dead (upper right: 7-AAD positive, apoptosis positive) cells. $^*P<0.05$, $^{***}P<0.001$ siEMCN vs siCtrl. Error bars represent SEM.

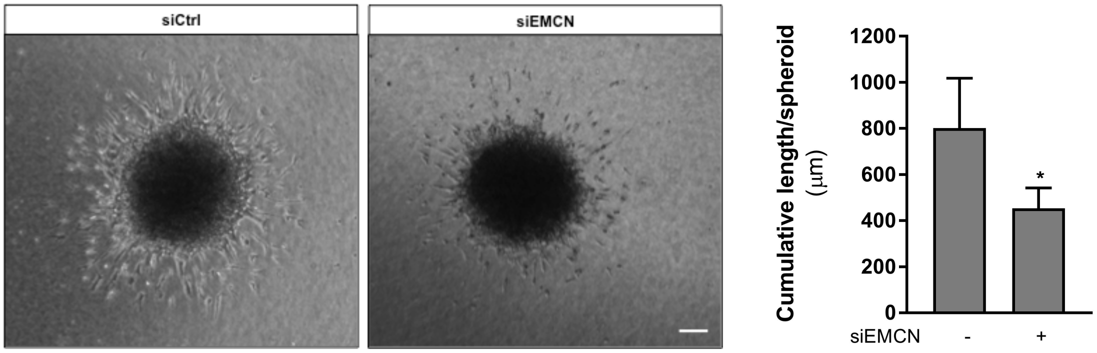


<u>Figure S3</u>: **EMCN knockdown attenuates capillary sprouting of endothelial cells in response to VEGF. (Left)** Representative images of angiogenic sprouting from spheroids generated from HRECs stimulated (12 hours) by VEGF (5 ng/ml). **(Right)** Quantification of total length of all sprouting processes originating from a single spheroid in the presence of VEGF (2-8 spheroids/well). Quantitative results are from three independent experiments. *$P$<0.05 siEMCN vs siCtrl. Error bars represent SEM. Scale bar, 100 μm.

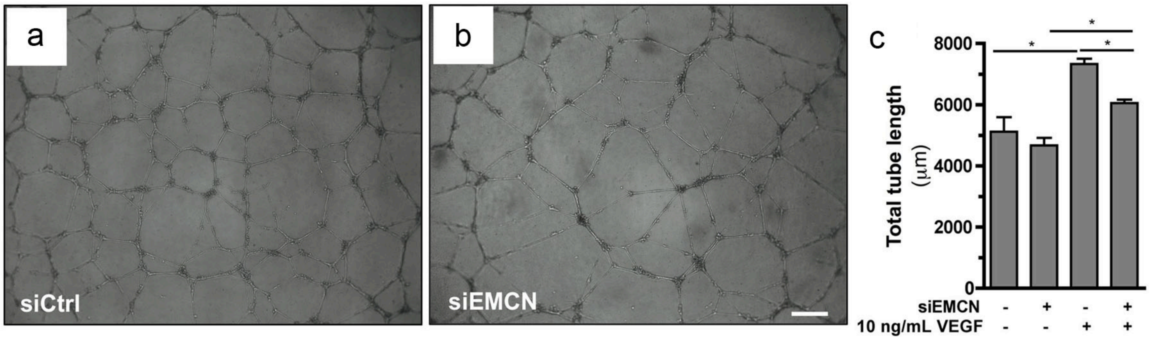


Figure S4: Reduced levels of EMCN render HRECs unable to organize into vessel-like structures at later time points. (a) siCtrl or (b) siEMCN cells were plated on polymerized BME in the presence of VEGF (10 ng/mL). Representative phase-contrast images after 24 hr are shown. (c) Quantification of total tube length was performed by Image J 24 hr after plating on BME in the presence and absence of VEGF shows knockdown of EMCN expression inhibits ECM-induced tube formation. Quantitative results are from three independent experiments. *$P$<0.05, ***$P$<0.001 siEMCN vs siCtrl or AdEMCN vs AdGFP; *$P$<0.05, **$P$<0.01 VEGF vs no VEGF. Error bars represent SEM. Scale bar, 100 μm.

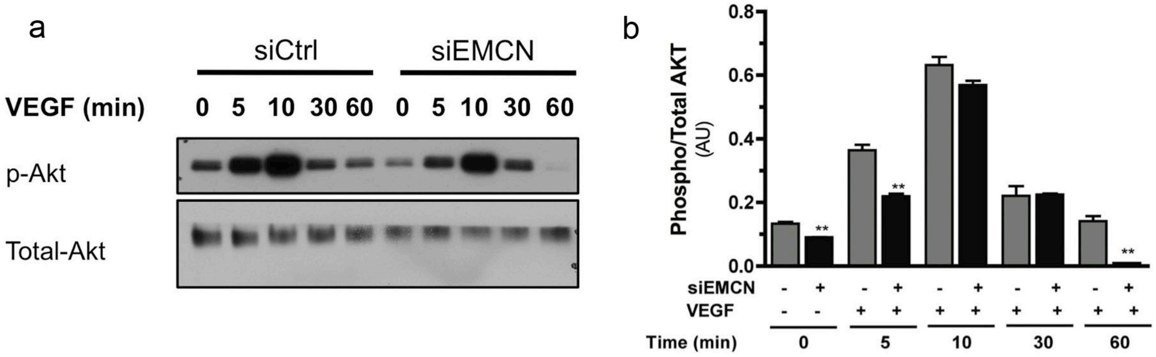


**<u>Figure S5</u>: EMCN inhibits VEGF-induced phosphorylation of Akt in HRECs. (a)** Immunoblot and **(b)** quantitative analysis of siCtrl and siEMCN cells stimulated with exogenous VEGF (10 ng/mL) at indicated time points and assessed for levels of phosphorylated and total Akt. ***P*<0.01. Error bars represent SEM. Cropped gels are displayed.
